# Relative roles of the different Pax6 domains for pancreatic alpha cell development

**DOI:** 10.1186/1471-213X-10-39

**Published:** 2010-04-09

**Authors:** Petra Dames, Ramona Puff, Michaela Weise, Klaus G Parhofer, Burkhard Göke, Magdalena Götz, Jochen Graw, Jack Favor, Andreas Lechner

**Affiliations:** 1 Ludwig-Maximilians-Universität, Klinikum Großhadern, Medizinische Klinik 2, Marchioninistr. 15, 81377 München, Germany; 2 Helmholtz Center Munich, German Research Center for Environmental Health, Institute for Stem Cell Research, Ingolstädter Landstraße 1, 85764 Neuherberg, Germany and Ludwig-Maximilians-Universität, Physiological Genomics, Schillerstr. 46, 80336 München, Germany; 3 Helmholtz Center Munich, German Research Center for Environmental Health, Institute of Developmental Genetics, Ingolstädter Landstraße 1, 85764 Neuherberg, Germany; 4 Helmholtz Center Munich, German Research Center for Environmental Health, Institute of Human Genetics, Ingolstädter Landstraße 1, 85764 Neuherberg, Germany

## Abstract

**Background:**

The transcription factor Pax6 functions in the specification and maintenance of the differentiated cell lineages in the endocrine pancreas. It has two DNA binding domains, the paired domain and the homeodomain, in addition to a C-terminal transactivation domain. The phenotype of Pax6^-/- ^knockout mice suggests non-redundant functions of the transcription factor in the development of glucagon-expressing α-cells as this cell type is absent in the mutants. We ask the question of how the differentiation of pancreatic endocrine cells, in particular that of α-cells, is affected by selective inactivation of either one of the three major domains of Pax6.

**Results:**

The Pax6^Aey18 ^mutant mouse line, in which the paired domain is inactivated, showed a phenotype similar to that of Pax6^-/- ^knockout mice with a near complete absence of glucagon-positive α-cells (0-4 cells/section; ≤1% of wt), reduced β-cell area (74% of wt) and disorganized islets. The proportion of ghrelin-positive ε-cells was expanded. In Pax6^Sey-Neu ^mutants, which lack the transactivation domain, α-and β-cells where reduced to 25 and 40% of wt, respectively. We also studied two mouse lines with mutations in the homeodomain, Pax6^4Neu ^and Pax6^132-14Neu^. Neighboring amino acids are affected in the two lines and both point mutations abolish DNA binding of the classical P3 homeodomain target sequence. The pancreatic phenotype of the two mutants however was divergent. While Pax6^4Neu ^homozygotes showed a reduction of α- and β-cells to 59 and 61%, respectively, pancreatic endocrine development was unaltered in the Pax6^132-14Neu ^mutant strain.

**Conclusions:**

We show that inactivation of the Pax6 paired domain leads to a more severe phenotype with regards to the differentiation of pancreatic α-cells than the loss of the transactivation domain. The analysis of two different homeodomain mutants suggests that the binding of Pax6 to P3 homeodomain consensus sequences is not required for α-cell development. It rather seems that the homeodomain has a modulating role in Pax6 function, possibly by facilitating a PH0-like binding confirmation on paired domain target genes like proglucagon. This function is differentially affected by the two homeodomain mutations analyzed in this study.

## Background

The development of the endocrine pancreas is governed by a cascade of transcription factors [1,2]. The first regulators are *Pdx1 *and *Ptf1a*, which initiate pancreatic bud formation from the foregut and ngn3, which separates the endocrine progenitor cells from the exocrine part of the organ. Subsequently, differentiation of the specialized mature endocrine islet cells is dependent upon specific sets of transcription factors for each cell type (reviewed in [3]).

Pax6 is one of the central regulators of specification and maintenance of the differentiated lineages within the pancreatic islet. It is expressed in the pancreas from embryonic day 9.5 exclusively in cells already committed to the endocrine tissue and it remains active postnataly in all islet cell types [4-6]. Pax6 also has major roles in the differentiation of the eye and the central nervous system [7]. It contains two DNA binding domains, the paired domain and the homeodomain, in addition to a C-terminal transactivation domain.

The pancreatic phenotype of Pax6^-/- ^knockout mice suggests that important and non-redundant functions are exerted by the transcription factor in glucagon-expressing α-cells as this cell type is absent in the mutants. Additional pancreatic changes are a reduced β-cell mass and disorganized islets [8]. The phenotype of inactivating Pax6 mutations (Pax6^Sey^, Pax6^Sey-Neu^), in which C-terminal deletions eliminate the homeodomain plus the transactivation domain or only the transactivation domain, respectively, is different from that of the knockout mice. In these mutant lines α-cells are present although reduced in numbers [9-11], which has also been interpreted as α-cell development being relatively normal with the exception of proglucagon gene expression itself [9]. The differing observations with knockout mice and Pax6^Sey/Sey-Neu ^mutants suggest that the different domains of Pax6 have diverse and independent functions in the specification of the endocrine cell lineages in the pancreas. Support for this hypothesis comes from the analysis of brain and eye development in different Pax6 mutant mouse lines. Here paired domain and homeodomain inactivation cause distinct phenotypes [7,12].

We ask the question of how the development of pancreatic endocrine cells, in particular that of glucagon-producing α-cells, is affected by selective inactivation of either one of the major domains of the transcription factor Pax6.

By analyzing different mutant mouse lines we found that inactivation of the paired domain results in a near complete loss of pancreatic α-cells while homeodomain and transactivation domain mutations led to normal or reduced α-cell numbers. Our results suggest a hierarchy in the roles of the three domains of Pax6 in pancreatic α-cell development. These findings could also help to reconcile the discordant observations previously reported for Pax6 domain-specific or knockout mutants.

## Results

### Loss of Pax6 paired domain but not of transactivation domain function leads to a near complete absence of fully differentiated α-cells

**Figure 1:**
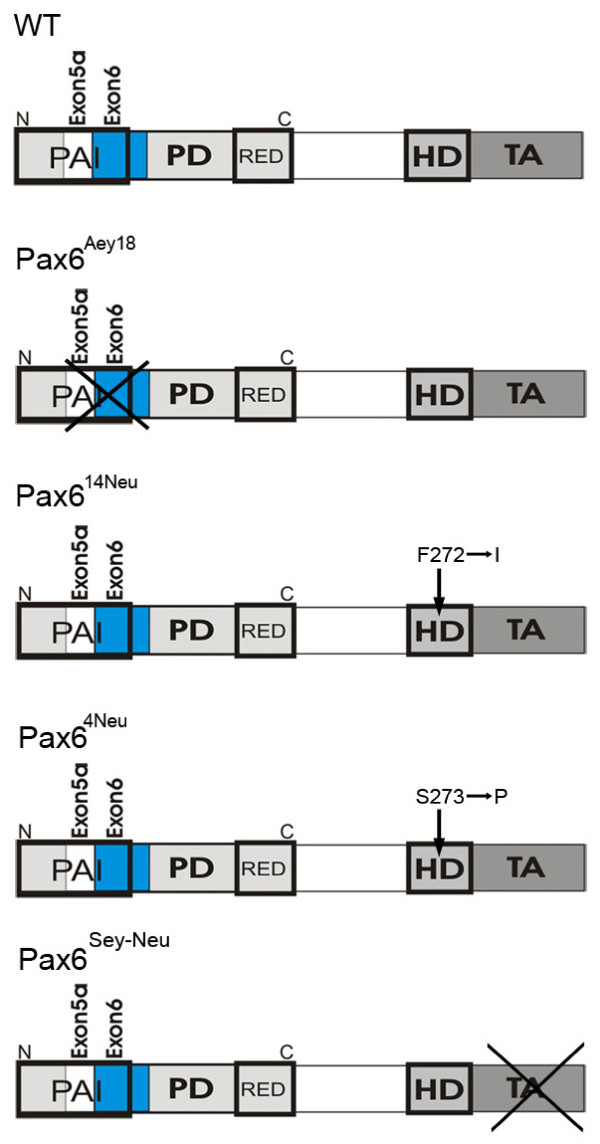
**Diagrams of the functional domains of Pax6 and of the alterations in the mutant mouse lines used in this study**. Pax6^Aey18 ^mutants lack exons 5a and 6 of the paired domain. Pax6^4Neu ^and Pax6^14Neu ^mutants carry point mutations at adjacent sites in the third helix of the homeodomain which abolish binding of that domain to its classical P3 DNA target sequence. Pax6^Sey-Neu ^lack the transactivation domain. (PD = paired domain; HD = homeodomain; TA = transactivating domain; PAI/RED = N- and C-terminal DNA-binding sub-domains of the PD).

To assess the functional role of the Pax6 paired domain in pancreatic endocrine development we studied the mutant mouse line Pax6^Aey18 ^in which a splice acceptor site in front of exon 6 is missing and consequently exons 5a and 6 are not included in the mature RNA. This deletion renders the PAI DNA-binding unit of the paired domain inactive. The homeodomain and the transactivation domain remain intact in this mutant (Figure 1) [13].

**Figure 2:**
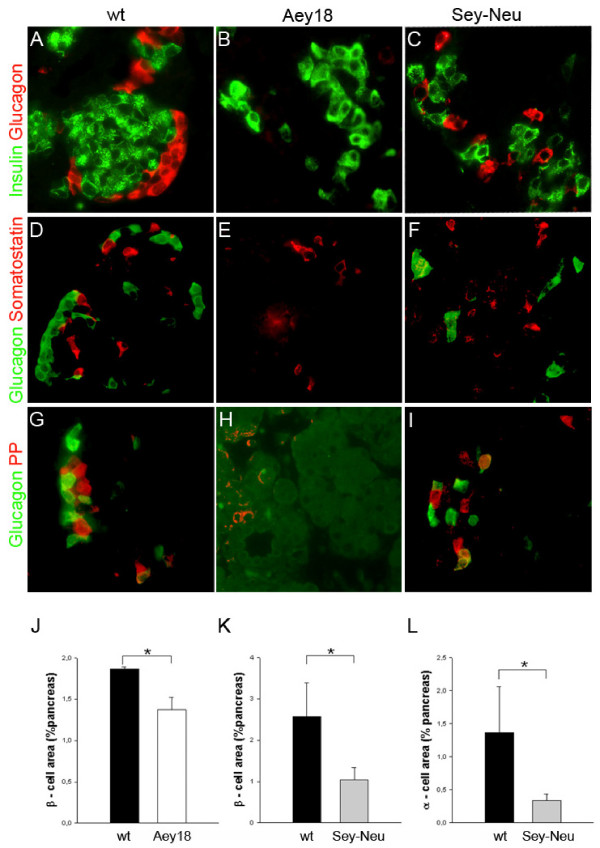
**Glucagon-expressing α-cells are more severely reduce in the paired domain mutant mouse line Pax6^Aey18^than in Pax6^Sey-Neu^homozygotes**. **A-I**. Immunstaining of pancreatic sections of e18.5 wt and mutant embryos. Glucagon^+ ^α-cells are nearly absent from the pancreas of paired domain mutant embryos (Pax6^Aey18^; 0-4 weakly positive cells/section; ≤1% of wt) but are present at 25% of wt in Pax6^Sey-Neu ^transactivation domain mutants. β-cells are reduced and islets are disorganized in both mutant lines. The proportion of somatostatin^+ ^δ-cells is visually unchanged in both mutants but PP cells are reduced in Pax6^Aey18^. **J-K**. Quantification of relative β-cell and α-cell area in wt and mutant e18.5 embryos. Individual cell sizes were comparable. (error bars = SD; 3-5 mice/group; * p < 0.05).

Like Pax6^-/- ^null homozygotes of Pax6^Aey18 ^die within hours after birth. We therefore analyzed the pancreas of e18.5 homozygotes in comparison to wild type (wt) littermates. Gross morphology of the pancreas was similar in both groups. However, we found that glucagon-producing α-cells were severely reduced in paired domain mutant embryos. Only 0-4 weakly glucagon-positive single cells per section were detected by immunostaining with the most sensitive of 3 different antibodies (≤1% of wt; Figure 2B). The area of insulin-producing β-cells was reduced to 74% of wt in the mutant animals (Figure 2J) while size of individual cells was unchanged (data not shown). Islet structure was disorganized (Figure 2B). Thus, the pancreatic phenotype of the Pax6^Aey18 ^paired domain mutants is similar to that of the Pax6^-/- ^knockout mice [8]. The proportion of somatostatin-positive δ-cells appeared unchanged but the number of PP cells also seemed reduced (Figure 2E, H). An exact quantification of these cells was not performed.

For comparison we also examined e18.5 pancreas of Pax6^Sey-Neu ^homozygous mutants. In these animals α-cells were present at 25% of wt animals (~50-100 cells/section) as was previously reported by others (Figure 2C, L) [10]. Also similar to previous findings, β-cells were reduced to 40% (Figure 2K) and islet structure was disorganized although less severe than in Pax6^Aey18 ^(Figure 2C) [10]. The proportion of δ- and PP cells appeared unchanged (Figure 2F, I).

### Paired domain inactivation not only affects proglucagon gene transcription but also results in an increase of ghrelin positive ε-cells and a reduced expression of prohormone convertase 2

The question has been raised whether Pax6 truly affects α-cell lineage commitment in the endocrine pancreas or whether it only directly targets proglucagon transcription [9]. To address this issue we first analyzed the distribution of the other endocrine cell types in the Pax6^Aey18 ^mutant pancreas.

**Figure 3:**
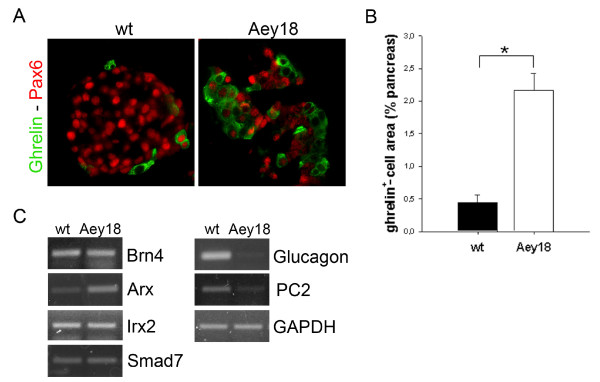
**Ghrelin^+ ^ε-cells are expanded and expression of prohormone convertase 2 is reduced in paired domain mutant embryos**. **A**. Immunstaining of pancreatic sections of e18.5 wt and Pax6^Aey18 ^mutant embryos. An increased number of ghrelin-expressing cells is seen in the paired domain mutants. These cells show variable degrees of Pax6 expression similar to the ε-cells in wt pancreas. **B**. ε-cells are expanded ~5fold in mutant animals. All ghrelin^+ ^cells were quantified regardless of their glucagon expression. Glucagon/ghrelin double-positive cells were present in wt animals but absent in Pax6^Aey18 ^homozygotes (data not shown). Thus the ghrelin^+ ^cells in the mutants can be classified as ε-cells. **C**. rtPCRs from total pancreatic RNA of e18.5 wt and Pax6^Aey18 ^mutant embryos. As expected, proglucagon expression is markedly reduced in the mutants. Of the other α-cell marker genes tested, only prohormone convertase 2 (PC2) shows a reduced expression level while the expression of Arx is increased.

We found an ~5fold expansion of ghrelin^+ ^cells (Figure 3A, B) compared to wt littermates. All ghrelin^+ ^cells were quantified in Figure 3B regardless of their glucagon expression. Glucagon/ghrelin double-positive cells were present in wt animals as previously described by others [6] (data not shown) but absent in Pax6^Aey18 ^homozygotes. Thus we classified the ghrelin^+ ^cells in the mutants as ε-cells. The quantity of additional ε-cells in the mutants was approximately equal to the reduction of α-cells, which indicates ghrelin expression in cells that are ghrelin^- ^in wt and could suggest a lineage switch from α- to ε-cells. A similar phenomenon has been described in Pax6^Sey ^mice [6]. Pax6 was expressed in ε-cells cells to various degrees, from undetectable to comparable to other islet cell types, both in mutant and wt pancreas (Figure 3A).

We also analyzed the expression of other relatively alpha cell specific genes in e18.5 Pax6^Aey18 ^pancreas compared to wt by rtPCR. We tested prohormone convertase 2 (pc2), which is a known Pax6 target [14] but in addition to alpha cells is also expressed in beta and cells [15] as well as Arx, Brn4, Irx2 and Gata6 for which evidence exists in the literature that they are quite alpha cell specific [9,16,17]., and Smad7, which favors alpha cell generation when overexpressed [18]. A significant reduction in expression in the mutants was only detected for pc2 in addition to the expected change in proglucagon transcription. Arx expression was increased in Pax6^Aey18 ^(Figure 3C).

Taken together the findings of ε-cell expansion and partially altered α-cell gene expression in paired domain mutant animals indicate a role of Pax6 in α-cell development that goes beyond proglucagon gene transcription. However, Pax6 probably functions late in lineage commitment, as the expression of major transcriptional regulators of α-cell development is not affected.

### The Pax6^Aey18 ^paired domain mutation (deletion of exon 6) leads to reduced nuclear localisation of Pax6 in vitro and in vivo

**Figure 4:**
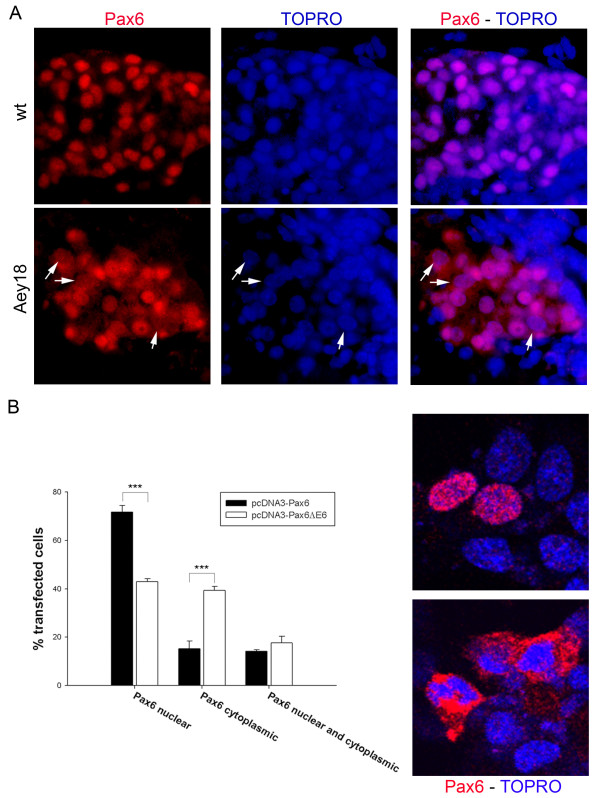
**The Pax6^Aey18 ^paired domain mutation (deletion of exon 6) leads to reduced nuclear localisation of Pax6 in vivo and in vitro**. **A**. Pancreatic endocrine cells of Pax6 wt embryos predominantly have a nuclear localization of Pax6 whereas it is found in the cytoplasm of many endocrine cells in Pax6^Aey18 ^paired domain mutants. In some cells, staining is predominantly cytoplasmatic (arrows) while others show equal cytoplasmatic and nuclear staining or largely exclusive nuclear staining similar to wt. **B**. To reconstruct the changes in the Pax6^Aey18 ^paired domain mutant mouse in vitro Exon 6 was deleted from an expression plasmid for canonical mouse Pax6. Wild type and mutated Pax6 where overexpressed in rat Ins1-E insulinoma cells. Cells where stained with anti-Pax6 and categorized into cells with pure nuclear, pure cytoplasmatic and mixed Pax6 localization. Only cells that clearly over-expressed the transcription factor over its endogenous level in Ins1-E cells where counted. After deletion of Exon 6 the percentage of cells with pure nuclear localization of Pax6 is substantially decreased (43% vs. 72% with wild type Pax6; *** p < 0,001). Representative confocal images of Ins1-E cells over-expressing Pax6 with either pure nuclear (wt) or pure cytoplasmatic localization (Pax6^Aey18^) of the protein are shown in the right panels.

Immunostaining of Pax6 in Pax6^Aey18 ^mutants revealed an altered intracellular distribution of the protein compared to wt. While Pax6 has an almost exclusive nuclear localization in wt increased cytoplasmatic immunoreactivity was observed in many mutant cells (Figure 4A). In some, staining was predominantly cytoplasmatic (arrows in Figure 4) while others showed equal cytoplasmatic and nuclear staining or largely exclusive nuclear Pax6 similar to wt. A comparable staining pattern was seen in the forebrain of mutant embryos (M.G., unpublished observation). In the Pax6^Aey18 ^mouse line the exons 5a and 6 are deleted. Since exon 5a is only included in one of the Pax6 isoforms we hypothesized that a nuclear localization signal (NLS) is located within exon 6 and that its absence results in enhanced cytoplasmatic sequestering of Pax6 in the Pax6^Aey18 ^mutants. We first performed a computational analysis of the Pax6 sequence with the PSORT II algorithm but found no classical NLS in any part of the protein, including exon 6. We therefore conducted an in vitro study to confirm a role of exon 6 in nuclear localization. Wild type Pax6 and the transcription factor with a deletion of exon 6 were transiently transfected into INS-1E insulinoma cells [19] serving as a model system for pancreatic endocrine cells. Using an antibody against the transactivation domain we determined the subcellular localization of the protein. We found a significant shift towards cytoplasmatic localization in cells transfected with Pax6ΔExon6 but no complete block of transfer into the nucleus (Figure 4B), similar to what is seen in vivo. Only cells that clearly over-expressed the transcription factor over its endogenous level where counted.

Exon6 therefore contributes to the nuclear localization of Pax6. However, since nuclear Pax6 is still detected in many cells this does not fully explain the phenotype of the Pax6^Aey18 ^mutant mice.

### Two neighboring point mutations in the homeodomain have differential effects on pancreatic endocrine development

We next analyzed the role of the second DNA binding domain of Pax6, the homeodomain, in the development of the endocrine pancreas. We examined two mutant mouse lines, Pax6^4Neu ^and Pax6^132-14Neu ^(Pax6^14Neu^), in which point mutations in the third helix of the homeodomain abolish the binding of that domain to the P3 homeodomain consensus DNA sequence [12,20]. Although neighboring amino acids are affected in the two mutants (Figure 1) Pax6^4Neu ^homozygotes die shortly after birth while Pax6^14Neu ^mutants live to adulthood and are fully fertile.

**Figure 5:**
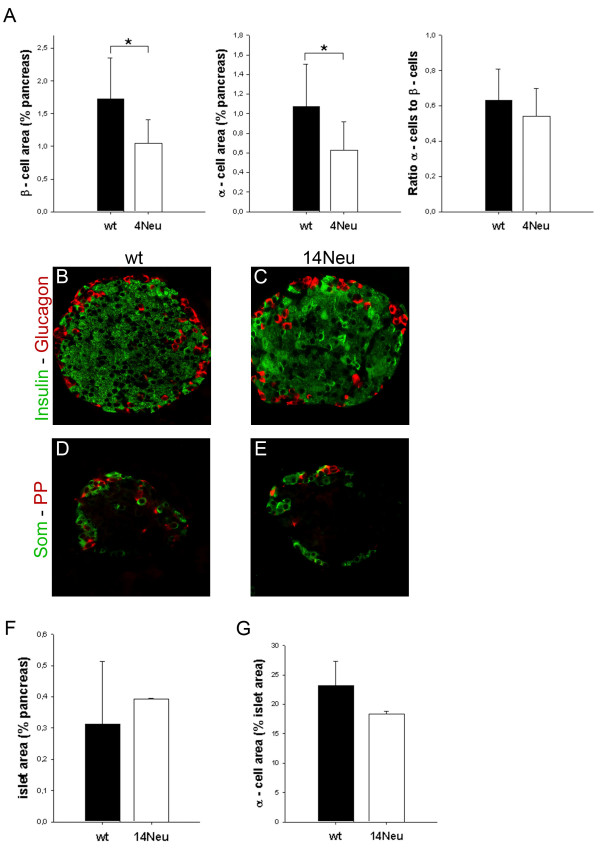
**The homeodomain has a modulating role on Pax6 function in the endocrine pancreas**. Two mutant mouse lines, Pax6^4Neu^and Pax6^14Neu^, in which point mutations in the third helix of the homeodomain abolish binding the P3 homeodomain consensus DNA sequence were studied. Although neighboring amino acids are affected in the two mutants Pax6^4Neu ^homozygotes die shortly after birth while Pax6^14Neu ^mutants live to adulthood. **A**. In e18.5 Pax6^4Neu ^homozygotes both α- and β-cells are reduced to 59 and 61 percent, respectively (6-7 animals/group; *p < 0,05). The ratio of α- to β-cells is unchanged. **B-E**. In 6 week old Pax6^14Neu ^homozygotes islet architecture is unchanged compared to wt. **F**. Islet- and α-cell area comparable in wt and Pax6^14Neu ^mutants (3 mice/group).

We studied e18.5 embryos homozygous for the Pax6^4Neu ^mutation and found that gross pancreatic morphology was unchanged in the mutant animals compared to wt. Quantification of both α- and β-cells revealed that they were reduced to 59 and 61%, respectively. The ratio of α- to β-cells remained unchanged (Figure 5A). We next examined 6 week old Pax6^14Neu ^homozygotes and compared them to wt controls. Fasting blood glucose was not significantly different (75 ± 5 vs. 61 ± 15 mg/dl; p = 0,33). Morphologically, islet architecture was unchanged (Figure 5B-E) in the mutants. Islet- and α-cell area also were comparable to wt controls (Figure 5F, G).

## Discussion

We address the question of whether the three major domains of the transcription factor Pax6, the paired domain, the homeodomain and the transactivation domain, have distinct roles in pancreatic endocrine development. We were particularly interested in glucagon-secreting α-cells, as that is the cell type that is absent in Pax6^-/- ^knockout mice [8].

We found that the pancreatic phenotype of the Pax6^Aey18 ^mutant mouse line, in which the paired domain is inactivated by a partial deletion, closely resembles that of the knockout mice. These mutants exhibit a near complete loss of glucagon-positive α-cells (≤1% of wt), a disorganized islet structure and a reduced β-cell area. A lack of Pax6 protein expression or protein degradation caused by the partial deletion of the gene can be excluded as a reason for this phenotype as Pax6 is clearly detected by immunostaining with an antibody against the transactivation domain. A general change in protein conformation, caused by the paired domain mutation, that contributes to the severe phenotype remains possible. It is however most likely that it is the inactivated paired domain directly that is responsible for the developmental alteration since loss of either of the other two major protein domains of Pax6 does not result in such a severe phenotype.

Endocrine development in Pax6^Sey-Neu ^mutants, that were often viewed as equivalent to Pax6^-/- ^knockouts, was strikingly different. In homozygotes, glucagon-positive α-cells were present at 25% of wt. This is in accordance with previous studies of Pax6^Sey ^and Pax6^Sey-Neu ^mutants [9,10]. Both strains share the loss of the transactivation domain but have an intact paired domain. The Pax6 knockouts on the other hand were generated by replacing the start codon and the entire paired domain with a beta-galactosidase neomycin cassette. No Pax6 protein was detected in these embryos [8]. Taken together we show that a functional Pax6 paired domain is more important for full α-cell development than the transactivation domain. Gene activation by Pax6 occurs in cooperation with other transcription factors, particularly large Mafs [21,22]. Both the paired domain and the homeodomain have been shown to independently facilitate at least a weak protein interaction [21]. It is therefore conceivable that in the Pax6^Sey-Neu ^mutants transcriptional activation, e.g. of the proglucagon gene, is achieved through the recruitment of other transcription factors to Pax6 binding sites. No compensation however seems possible for the loss of paired domain function suggesting that in the pancreas this domain acts as a major DNA anchor for transcriptional activator complexes involving Pax6. The finding by Sander et al. [10] that no immunreactive Pax6 can be detected in the pancreas of Pax6^Sey-Neu ^homozygous embryos with an antiserum raised against the paired domain is difficult to reconcile with the presence of a substantial number of α-cells in these mutants. In light of our findings and the knockout phenotype some residual Pax6 seems necessary for glucagon-positive cells to develop to the extent seen in these mutants. Possible explanations could be an expression level below the sensitivity of immunostaining or conformational changes in the Pax6^Sey-Neu ^protein that diminish the binding of the antiserum.

The proglucagon gene is a direct target of Pax6, which binds to the G1 and G3 elements of the gene promoter, mainly via the paired domain [22]. Thus the question arises whether Pax6 is truly involved in the lineage commitment of α-cells or whether the phenotype upon Pax6 inactivation is only the result of impaired glucagon expression in otherwise fully differentiated α-cells [9]. We addressed this issue by first looking at changes in other endocrine lineages and also by analyzing the expression of other genes related to α-cell differentiation. We found an ~5fold increase in the number of ghrelin-positive ε-cells in the paired domain mutants. A similar observation has been reported previously for Pax6^Sey ^[6] and also for Nkx2.2 null mice [23]. The expansion of ε-cells in our study is approximately equal to the reduction in the number of α-cells. This suggests a lineage switch from α- to ε-cells although we do not provide direct evidence for that. One possible alternative explanation could be a β-cell to ε-cell switch as β-cells are also significantly reduced in the mutant mice. We also analyzed a number of relatively α-cell specific genes in the paired domain mutants but only found a reduction in the expression of proglucagon and prohormone convertase 2. Both genes are known targets of Pax6 [14] but pc2 is also expressed in beta and cells, in addition to alpha cells [15]. Expression of the relatively α-cell specific transcription factors Brn4 and Irx2 was unchanged, while the expression of Arx was increased. Overall our results indicate that the developmental phenotype after Pax6 inactivation is more extensive than only an absent proglucagon transcription but that the disruption of α-cell development occurs at a late stage downstream of Brn4, Irx2 and Arx. This in accordance with previous findings [9,16]. The observed increase in Arx expression could be the result of a feedback loop but experimental evidence for this hypothesis is lacking. Similarly, it is currently unknown how the likely de-repression of ghrelin occurs in the different Pax6 mutants.

We also found that a deletion of exon 6, as in Pax6^Aey18^, leads to increased cytoplasmatic localization of the transcription factor although no classical NLS is present in this sequence. Our observation however is in line with a previous analysis of naturally occurring splice variants of the avian homologue of Pax6, Pax-QNR [24]. The reduced nuclear localization of Pax6 might add to the phenotype of the Pax6^Aey18^mutants. However, it does not by itself explain the near complete absence of α-cells since nuclear Pax6 at the level of wt controls is still detectable in a proportion of endocrine cells in the mutant embryos.

The homeodomain of Pax6 seems to have a modulating effect on pancreatic endocrine development. For classical homeodomain function, dimers of homeodomain transcription factors bind to a palindromic target sequence, e.g. P3 in the case of Pax6 [25]. We analyzed two homeodomain mutants, Pax6^4Neu ^and Pax6^14Neu^. Neighboring amino acids are mutated in the two lines and both point mutations abolish binding to the P3 homeodomain target sequence [12,20]. However, activation of a proglucagon promoter fragment containing the G1 element is differentially affected by the two mutations. Pax6^4Neu ^shows a decreased function compared to wt while the activity of Pax6^14Neu ^is slightly increased [12]. We found that Pax6^4Neu ^mutants, which die shortly after birth (slower than homozygous null mutants), show a reduced endocrine cell mass with both α and β-cells affected to a similar extent. Pax6^14Neu ^homozygotes that develop to adulthood on the other hand had normal islet morphology, unchanged islet- and α-cell areas and normal fasting blood glucose. These observations suggest that independent homeodomain binding of Pax6 to P3 elements is of little importance in the development of the endocrine pancreas although a detailed analysis of glucose metabolism in adult Pax6^14Neu ^mice has yet to be done. Our findings rather indicate that the homeodomain co-activates paired domain target genes and that this is impaired by the Pax6^4Neu ^but not by the Pax6^14Neu ^mutation. This is in line with the activity of both mutated proteins on the proglucagon promoter [12]. A possible explanation could be that the homeodomain facilitates a PH0-like binding confirmation [26] of Pax6 on target genes relevant for pancreatic endocrine development. This binding confirmation has already been demonstrated for the rat proglucagon promoter G1 and G3 sites [26,27].

## Conclusions

We demonstrate in this study that the paired domain of Pax6 is more important for the full differentiation of glucagon-producing pancreatic α-cells than its transactivation domain. Independent DNA binding of the Pax6 homeodomain seems least relevant in the pancreas. The homeodomain rather appears to have a modulating role in the function of the transcription factor, possibly by facilitating a PH0-like binding confirmation on paired domain target genes.

## Methods

### Animals

The mutant mouse lines Pax6^Aey18^, Pax6^Sey-Neu^, Pax6^4Neu ^and Pax6^132-14Neu ^were identified in mutagenesis experiments based on their eye phenotype and subsequently identified as Pax6 mutant alleles by linkage analysis and a positional candidate gene approach [13,20,28]. The mice were maintained on a C3HeB/FeJ background. Analysis was performed with embryos from embryonic day (e)18.5 and 6 week old mice (Pax6^132-14Neu^). All animal studies were done in accordance with governmental and institutional regulations.

### Antibodies

Guinea pig anti-insulin (Daco) 1:1000; Guinea pig anti-glucagon (Linco) 1:1000; Rabbit anti-glucagon (Dako) 1:500; Rabbit anti-Glucagon (Chemicon) 1:500; Goat anti-ghrelin (Sant Cruz) 1:200; Rabbit anti-pax6 (Chemicon) 1:2500; Rabbit anti-somatostatin (Chemicon) 1:100; Rabbit anti-pancreatic polypeptide (Chemicon) 1:30

### Tissue preparation and immunostaining

Tissues were fixed in 4% buffered formalin for 24 hours, embedded in paraffin and cut into 4 μm sections. For immunostaining sections were dewaxed in xylene, hydrated, incubated for 30min 0.01mol/l Na-citrate buffer in a steamer for antigen retrieval, washed in PBS and blocked with 1% normal donkey serum in PBS containing 0.1% Triton. They were then incubated with the first antibodies at 4°C over night, washed 3× in PBS, incubated with Cy2 and Cy3 labelled secondary antibodies (Jackson Immuno Research), washed again 3× in PBS and mounted with fluorecence mounting media. Nuclear counterstaines were done with DAPI or TO-PRO^®^-3 iodide (both pseudocolored blue in multicolor images). Images were captured with a CCD camera attached to a Zeiss Axioscope and also a Leica laser scanning microscope.

### Quantification of α-, β-, ε-cell and islet area

For cell quantification tissue sections (2-3/embryo) were stained with the respective antibodies. The total area of pancreatic tissue and the total area of either hormone positive cells or whole islets were measured on digital images of each section using ImageJ. The relative α-, β-, ε-cell and islet area was determined by division by the total pancreatic area on each section. Student's t-Test was used to calculate statistical significance.

### rtPCR

Embryonic pancreas was detached from other tissue under a dissection microcope. Total RNA was isolated using the RNeasy kit (Qiagen) including the removal of genomic DNA. cDNA was generated using the ImProm-II™ Reverse Transcription System (Promega). Samples were normalized for GAPDH expression. The minimal number of PCR cycles was used for each gene product. -RT controls were done for all reactions and the amplification of the correct cDNAs was confirmed by sequencing individual PCR products.

### Overexpression of Pax6 and Pax6ΔExon6 in vitro

The complete coding sequence of canonical Pax6 was cloned into the pcDNA3 expression vector. Deletion of Exon6 was achieved by site directed mutagenesis using the Phusion^® ^Site-Directed Mutagenesis Kit (New England Biolabs; forward primer: GTGTCATCAATAAACAGAGTTCTTCGCA; reverse primer: CTGCAGAATTCGGGAAATGTCGCAC). Vector constructs were sequenced to confirm the correct identity of the expressed cDNA. Transfection into INS-1E insulinoma cells seeded on glass cover slips was done with Rotifect (Roth). 72 hours after transfection cells were fixed in 4% PFA and immunostained as described for tissue sections. Nuclear counterstaining was done with TO-PRO^®^-3 iodide (Invitrogen; pseudocolor blue in Figure 5). 100 transfected cells per sample were counted for quantitative analysis. Student's t-Test was used to calculate statistical significance.

## Authors' contributions

PD, RP and MW did the experimental work in the laboratory, KGP and BG helped review the data, PD and AL analyzed the data and wrote the paper, MG helped with immunostaining and data analysis, JF and JG maintained the mutant mouse lines and helped with identification of homozygotes, tissue preparation and writing the paper. All authors read and approved the final manuscript.
